# *Potentilla chinensis* aqueous extract attenuates cyclophosphamide-induced hemorrhagic cystitis in rat model

**DOI:** 10.1038/s41598-022-17393-8

**Published:** 2022-07-29

**Authors:** Kajetan Juszczak, Jan Adamowicz, Łukasz Zapała, Tomasz Kluz, Przemysław Adamczyk, Artur Wdowiak, Iwona Bojar, Marcin Misiek, Magdalena Emilia Grzybowska, Klaudia Stangel-Wójcikiewicz, Ewa Poleszak, Marta Pokrywczyńska, Tomasz Drewa, Andrzej Wróbel

**Affiliations:** 1 Department of Urology and Andrology, Collegium Medicum, Nicolaus Copernicus University, M. Curie Skłodowskiej 9, 85-094 Bydgoszcz, Poland; 2 Department of Regenerative Medicine, Collegium Medicum, Nicolaus Copernicus University, Bydgoszcz, Poland; 3 Clinic of General, Oncological and Functional Urology, Medical University of Warsaw, Warsaw, Poland; 4 Department of Gynecology and Obstetrics, Institute of Medical Sciences, Medical College of Rzeszow University, Rzeszow, Poland; 5 Department of General and Oncological Urology, Nicolaus Copernicus Hospital, Torun, Poland; 6 Chair of Obstetrics and Gynecology, Faculty of Health Sciences, Medical University of Lublin, Lublin, Poland; 7 Department of Women’s Health, Institute of Rural Health in Lublin, Lublin, Poland; 8 Department of Gynecologic Oncology, Holy Cross Cancer Center, Kielce, Poland; 9 Department of GynecologyGynecological Oncology and Gynecological Endocrinology, Medical, University of Gdańsk, Gdańsk, Poland; 10 Department of Gynecology and Oncology, Jagiellonian University Medical College, Cracow, Poland; 11 Chair and Department of Applied and Social Pharmacy, Laboratory of Preclinical Testing, Medical University of Lublin, Lublin, Poland; 12 Second Department of Gynaecology, Medical University of Lublin, Jaczewskiego 8, 20-954 Lublin, Poland

**Keywords:** Bladder, Toxicology

## Abstract

Cyclophosphamide (CYP) damages all mucosal defence lines and induces hemorrhagic cystitis (HC) leading to detrusor overactivity. Patients who undergo combined chemio-radiotherapy are at higher risk of HC. *Potentilla chinensis* extract (PCE) prevent oxidative stress-dependent diseases. Thus, the aim of the study was to investigate the effect of PCE on urinary bladder function in CYP-induced HC in preclinical study. 60 rats were divided into 4 groups, as follows: I—control, II—rats with CYP-induced HC, III—rats received PCE in dose of 500 mg/kg, and IV—rats with CYP-induced HC which received PCE in dose of 500 mg/kg. PCE or vehicle were administered orally for 14 days. The cystometry was performed 3 days after the last dose of the PCE. Next, urothelium thickness and oedema measurement and biochemical analyses were performed. Cyclophosphamide induced hemorrhagic cystitis. PCE had no influence on the urinary bladder function and micturition cycles in normal rats. PCE diminished the severity of CYP-induced hemorrhagic cystitis. In the urothelium the cyclophosphamide induced the elevation of CGRP, TNF-α, IL-6, IL-1β, OTC_3,_ NIT, and MAL. Also, the level of T-H protein, HB-EGF, and ZO1 was decreased. Moreover, the level of ROCK1 and VAChT in detrusor muscle increased. cyclophosphamide caused an increased concentration of BDNF and NGF in the urine. In turn, PCE in cyclophosphamide-induced hemorrhagic cystitis caused a reversal of the described biochemical changes within urothelium, detrusor muscle and urine. PCE attenuates detrusor overactivity. In conclusion, our results revealed that PCE attenuates detrusor overactivity in case of cyclophosphamide-induced hemorrhagic cystitis. The potential properties of PCE appear to be important in terms of preventing of oxidative stress-dependent dysfunction of urinary bladder. PCE may become a potential supportive treatment in patient to whom cyclophosphamide-based chemotherapy is used.

## Introduction

Chemotherapy based on oxazophorine alkylating agents (cyclophosphamide—CYP or ifosfamide) and radiotherapy in the area of urinary bladder play role in hemorrhagic cystitis (HC) pathogenesis. Patients who undergo combined chemio-radiotherapy are at higher risk of HC development^1^. CYP treatment damages all mucosal defence lines and induces chemical, hemorrhagic, cystitis leading to detrusor overactivity in animals and humans. The responsible key factor is acrolein, an irritant metabolite of CYP and ifosfamide, eliminated in the urine^2,3^. A number of prevention strategies in HC development have been tested. However, only the agents which binds to acrolein (e.g. Mesna) are widely used in clinical practice to limit the CYP-induced damage of urinary bladder^4^. Barunt et al.^5^ study revealed that ambroxol improve cyclophosphamide-induced hemorrhagic cystitis via antioxidant and antiinflammatory properties. Moreover, glycyrrhetinic acid liposomes provide benefits against cyclophosphamide-induced cystitis, which possibly occurs through underlying mechanisms that inhibit cell death and inflammatory stress^6^. Previous studies reveled that irradiation triggers ROS/RNOS-dependent pathway and/or oxidative/nitrosative-based stress leading to chronic inflammation and in a consequence involved organ dysfunction. Moreover, tissue damage induced by irradiation may be caused by increased production of pro-inflammatory cytokines^7^.

Herbal remedies are characterized by antioxidant properties. *Potentilla chinensis* extract (PCE) contains polyphenols which present antioxidative effects. These properties of PCE appear to be important in terms of preventing of oxidative stress-dependent diseases development^8,9^. Previous studies described several bioactive compounds which were extracted from *Potentilla chinensis*. Qiao et al.^10^ study, revealed that trans-tiliroside, a constituent from *Potentilla chinensis*, presents an anti-hyperglycemic, anti-hyperlipidemic and antioxidant activities. Qiu et al.^11^ isolated from *Potentilla chinensis* different polysaccharides (PCPW, PCPS1 and PCPS2) with immunological activity via activation of the NF-κB. Additionally, several chemical constituents of Potentilla chinesis (triterpenes) show anticancer activity^12^. Herbal drugs may become a potential supportive treatment in addition to typical pharmacotherapy with proven effect in daily clinical practice. Up till now herbal remedies are not routinely used in the treatment of urinary bladder dysfunction due to chemotherapy and/or radiotherapy. Thus we tested the effect of PCE on urinary bladder function in naïve condition, as well as in CYP-induced hemorrhagic cystitis.

Therefore, the purpose of the study was to investigate the attenuation effect of *Potentilla chinensis* aqueous extract on urinary bladder function in CYP-induced hemorrhagic cystitis in rat model, and thus to evaluate if *Potentilla chinensis* aqueous extract could be a valuable natural pharmacological agent in the treatment of urinary bladder dysfunction in patients after CYP-based therapy.

## Material and methods

### Animals

The experiment was performed on sixty female Wistar rats (weight: 200–225 g, age: 10 weeks). All rats were housed individually in the metabolic cages (3700M071, Tecniplast, West Chester, PA, USA). The living condition of rats were constant (temperature: 22–23 °C/humidity: 45–50%/day-night cycle: 12:12 h). All rats were fed with animal food without any restriction to water. All experimental procedures were conducted in accordance with the European Communities Council Directive of 22 September 2010 (2010/63/EU). The experimental protocol was approved by the Local Ethics Committee. The sample size calculation was performed using G*Power ver. 3.1.9.6 software. For the power level of 0.95, medium group effect (f = 0.25) and provided 4 experimental were subjected to ANOVA testing, the total number of 60 rats was proposed to the experiment.

### Experimental groups

Sixty rats were divided randomly into four groups of fifteen animals each, as follow:

- Rats received saline [group I: control group (n = 15)],
- Rats with cyclophosphamide (CYP)-induced DO [group II: CYP (n = 15)],
- Rats received PCE in dose of 500 mg/kg [group III: PCE (n = 15)],
- Rats with CYP-induced DO who received PCE in dose of 500 mg/kg [group IV: CYP + PCE (n = 15)].

### Study protocol

The *Potentilla chinensis* aqueous extract or vehicle were administered orally for the period of 14 days after surgical procedure. Cystometry was performed 3 days after the last dose of the PCE administration (in 17th day after urinary bladder catheter implantation). After cystometric evaluation the rats were killed by decapitation for further evaluation. The urothelium thickness measurement, assessment and biochemical analyses were performed.

### Drugs

The following drugs were used: (1) cyclophosphamide (CYP; Endoxan, Baxter Deutschland GmbH, Unterschleiβheim, Germany). CYP was diluted in a physiological saline (0.9% NaCl) and administered intraperitoneally (i.p.) as a single dose of 200 mg/kg, as described previously^13,14^, (2) *Potentilla chinensis* extract (PCE) was purchased from SK Bioland (Cheonan, South Korea). The yield of aqueous extract was 21%. PCE was administered orally at a single daily dose of 500 mg/kg for 14 days as previously described^15^. Similarly the control animals received volume-matched dose of vehicle.

### Surgical procedures

All the surgical procedures were performed under intraperitoneal, combined-anaesthesia using Ketamine hydrochloride (dose: 75 mg/kg) (Ketanest; Pfizer, Warsaw, Poland) and Xylazine (dose: 15 mg/kg) (Sedazin; Biowet, Puławy, Poland), as described previously^15^. Before surgical procedure cefazolin sodium hydrate (Biofazolin; Sandoz, Warsaw, Poland) given subcutaneously with 100 mg dose were used for prevention of urinary tract infection. All animals were positioned on supine position on a warming mattress (37 °C) before surgical procedure. The proper depth of anaesthesia was reached when no spontaneous movement and no withdrawal response to a noxious toe pinch were observed. After proper anaesthesia, the shaved and cleaned abdominal wall was opened through an approx. 10-mm vertical midline incision. The urinary bladder was gently freed from the adherent tissues. A double lumen polyethylene catheter (inside diameter—i.d.: 0.28 mm and outside diameter—o.d.: 0.61 mm; BD, Franklin Lakes, NJ, USA), filled with physiological saline with a cuff at the end was inserted into urinary bladder through a small incision into the apex of the urinary bladder and sutured with a 6–0 Vicryl stitch. Moreover, during the same session, in order to measure the blood pressure, the carotid artery was cannulated with a polyethylene catheter (i.d.:0.28 mm and o.d.: 0.61 mm; BD) filled with 40 IU/ml heparinised physiological saline. The catheters were tunnelled subcutaneously and exteriorised in the retroscapular region. The catheters were connected with a plastic adapter, to avoid the risk of removal by the animal. The chronically implanted intravenous catheter ensured stress-free conditions during the experiment. Additionally, to prevent form adhesions development and excessive fibrotic tissue production, the elastoviscous hyaluronan solutions—Healon (Pharmacia A.B.) at a dose of 0.85 ml was applied around the urinary bladder. After catheter implantation the abdominal wall was closed in multiple layers using 4/0 catgut sutures. The free ends of the catheters were sealed with silk ligatures.

### Conscious cystometry

Cystometry was performed 17 days after the surgical procedures (i.e. 3 days after the last dose of PCE administration), as previously described^16,17^. The urinary bladder catheter was connected via a three-way stopcock to a pressure transducer (FT03; Grass Instruments) situated at the level of the urinary bladder and to a microinjection pump (CMA 100; Microject, Solna, Sweden) for recording intravesical pressure and for constant saline infusion into urinary bladder. Conscious cystometry was performed by slowly filling of saline at a constant rate of 0.05 ml/min and at room temperature (22 °C) to elicit repetitive voiding. The infusion rate was based on pilot studies in which the rate of 0.05 to 0.1 ml/min was associated with urinary bladder cystometry profiles similar to those in the intact lower urinary tract in rats. The analogue signal obtained from the pressure transducer was amplified and digitised using the Polyview system (Grass Instruments). Micturition volumes were measured by means of a fluid collector attached to a force displacement transducer (FT03C; Grass Instruments). Both transducers were connected to a polygraph (7 DAG; Grass Instruments). Cystometry profiles and micturition volumes were recorded continuously on a Grass polygraph (Model 7E; Grass Instruments) and were determined graphically. The data were analysed using a sampling rate of 10 samples/s. The measurements in each animal represent the average of five micturition cycles after obtaining repetitive voiding. The mean values from all animals in each condition were averaged to create pooled data for each condition. All procedures were performed by a person blinded to the treatments. The following cystometric parameters were analyzed: (1) basal pressure (BP, cmH_2_O): the lowest urinary bladder pressure during filling phase (in animals with zero residual volume this is the passive pressure in the empty bladder and in animals with residual volume it corresponds to the pressure at this volume), (2) threshold pressure (TP, cmH_2_O): urinary bladder pressure immediately before onset of micturition contraction, (3) micturition voiding pressure (MVP, cmH_2_O): maximum urinary bladder pressure during micturition. As the rats do not seem to strain and have an abdominal pressure close to zero, micturition pressure is almost identical to the detrusor pressure, (4) intercontraction interval (ICI, sec): interval between MVP and the next MVP, (5) urinary bladder compliance (BC, ml/cmH_2_O): urinary bladder compliance was calculated as the bladder capacity divided by the difference in the pressure threshold and baseline pressure using the formula [(VV + PVR)/(TP − BP)]; It is routinely measured as an index of bladder storage function. Since the bladder can be extended at lower intra-bladder pressure, higher compliance indicates a better storage function, (6) voided volume (VV, ml): volume of expelled urine, (7) post-void residual (PVR, ml): urinary bladder capacity minus voided volume/fluid remaining in the urinary bladder at the end of micturition, (8) volume threshold to elicit NVC (VTNVC, %)—percent of total bladder filling volume, which is the preclinical equivalent to the volume at first involuntary detrusor contraction measured during urodynamic investigations in humans, (9) nonvoiding contractions (NVCs): frequency (FNVC, times/filling phase) and amplitude (ANVC, cmH_2_O)—an increase in urinary bladder pressure without release of fluid from the urethra. Non-voiding contractions (urinary bladder pressure increases before each micturition without the expulsion of the fluid) higher than 2 cmH_2_O were used as a surrogate for detrusor overactivity^18,19^. A voiding contraction was identified as a large increase in bladder pressure accompanied by the release of fluid from the urethra, (10) area under curve (AUC): defined the area under the cystometrogram trace reflecting the motility activity of the detrusor—we used total AUC method which determines area under the curve^13^, (11) detrusor index (DI, cmH_2_O/ml) (in group I and III) and detrusor overactivity index (DOI, cmH_2_O/ml) (in group II and IV): depicted as a quotient of the sum of amplitudes of all detrusor contractions during the filling phase (all NVCs and micturition voiding pressure) and functional bladder capacity. During calculation of DOI, the baseline bladder pressure (basal pressure at the beginning of the detrusor contraction) was subtracted from the height (absolute value of the contraction—from 0 cmH_2_O) of each detrusor contractions (each NVCs and micturition voiding pressure)^9,15^.

### Urothelium thickness measurement

The measurement of urothelium thickness was carried out as previously described^20^. The image analyzer computer system Leica Qwin 500 Image Analyzer (Leica Imaging Systems Ltd., Cambridge, England) was used to evaluate the urothelium thickness in micrometer using the interactive measure menu and hematoxylin and eosin-stained sections. A mean of 15 readings was estimated from five serial sections from slides of each animal in each group using low magnification (× 10).

### Urinary bladder wall oedema assessment

The evaluation of urinary bladder wall oedema was conducted by assessing vesical vascular permeability with the Evans Blue dye leakage technique, as previously described^13,14^. Catheter was inserted in the right femoral vein and Evans Blue (dose: 50 mg/kg) was injected intravenously. 30 min after Evans Blue supply, the animals were sacrificed and the urinary bladders were excised and weighed, which were then cut lengthwise and placed in a volume of 1 ml of formamide solution at 56 °C for 24 h. Formamide absorbance analysis was performed at 620 nM comparing it to a standard curve. The obtained results were expressed in nanograms of Evans Blue per milligram of the urinary bladder.

### Biochemical evaluation

In the urothelium of urinary bladder the following biomarkers were analysed: calcitonin gene related peptide (CGRP; Biomatik, CN EKU02858), tumor necrosis factor alpha (TNF-α; LifeSpan BioSciences;nLS-F5193), interleukin 6 (IL-6; LifeSpan BioSciences; LS-F25921), interleukin IL-1β (IL-1β; Cloud-Clone; SEA563Ra), organic cation transporter 3 (OCT_3_; Antibodies-online, CN ABIN6227163), Tamm–Horsfall protein (T–H protein, uromodulin; Antibodies-online; CN ABIN855058), heparin-binding epidermal growth factor-like growth factor (HB-EGF; Biomatik; CN EKU04742), 3-nitrotyrosine (NIT; LifeSpanBioSciences; CN LS-F40120-1), malondialdehyde (MAL; Biomatik, CN EKF57996), tight junction protein 1 (ZO1; CUSABIO, CSB-E17287r). Moreover, the level of Rho kinase (ROCK1; LifeSpan BioSciences, LS-F32208) and vesicular acetylcholine transporter (VAChT; LifeSpan BioSciences, CN LS-F12924-1) were determined within detrusor muscle of the urinary bladder. Additionally the urine samples were collected for the estimation of the following biomarkers: brain-derived neurotrophic factor (BDNF; PROMEGA, CN G7610) and nerve growth factor (NGF; LifeSpan BioSciences, CN LS-F25946-1). The protein concentrations in biological fluids are most common measured using enzymatic immunoassay ELISA^21^. The evaluation of biomarkers concentration in urothelium, detrusor muscle and urine was performed using enzyme-linked immunosorbent assay (ELISA) according to the manufacturers’ instructions, as previously described^14,22^. Each sample was measured in duplicate.

### Statistical analysis

All data were assessed by the one-way analysis of variance (ANOVA) followed by a Tukey *post-hoc* test (Statistica, v. 10, StatSoft, Inc., Tulsa, OK, USA). All results are presented as the mean ± standard error of the mean (SEM). p < 0.05 was considered as statistically significant with 95% confidence.

### Ethical approval

Ethical approval to report this case was obtained from The Polish Local Ethics Committee in Lublin, Poland (no. 323/20/UML).

### Statement of human and animal rights

All procedures in this study were conducted in accordance with The Polish Local Ethics Committee in Lublin, Poland (no. 323/20/UML) approved protocols. This study was carried out in compliance with the ARRIVE guidelines.

## Results

### Biological profile of experimental groups

**Figure 1:**
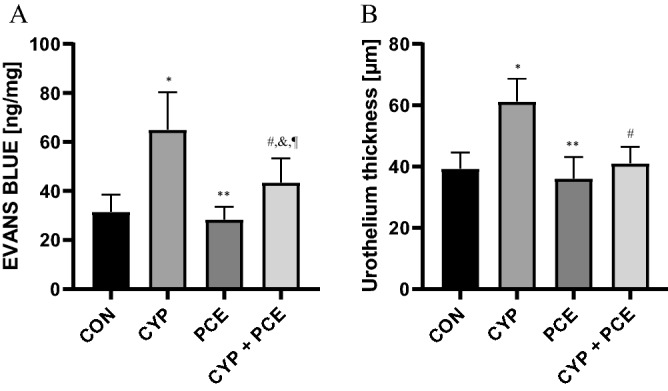
The influence of cyclophosphamide (CYP) and *Potentilla chinensis* aqueous extract (PCE) in naïve rats and PCE in rats with CYP-induced cystitis on the level of (**A**) bladder wall oedema, and (**B**) urothelium thickness.

CYP treatment causes mucosal inflammatory response as indicated by macroscopic changes within urinary bladder. The CYP administration increased the urothelium thickness (n = 15). In contrast, the supply of PCE prevented the excessive thickening of urothelium in response to CYP (n = 15). The urinary bladder wall oedema was particularly noticeable. In the response to CYP the severity of urinary bladder wall oedema was significantly higher as compared to naive rats (n = 15). Moreover, PCE intake attenuated the severity of oedema in rats with CYP-induced HC (n = 15) (Fig. 1, Table 1).

**Table 1:** Biological parameters of experimental groups.

Biological parameters	Experimental groups				p
	I control (n = 15)	II CYP-induced DO (n = 15)	III PCE (n = 15)	IV CYP-induced DO + PCE (n = 15)	
UT (µm)	39 ± 1.4	61 ± 1.9*	36 ± 1.8^§^	41 ± 1.4^#^	p < 0.0001* p < 0.0001^§^ p < 0.0001^#^
EVANS BLUE (ng/mg)	32 ± 1.8	65 ± 4.0*	28 ± 1.3^§^	43 ± 2.5^#,&,¶^	p < 0.0001* p < 0.0001^§^ p < 0.0001^#^ p = 0.0104^&^ p = 0.0008^¶^

*Statistically significant differences between group I and group II (p ≤ 0.05), ^§^Statistically significant differences between group II and group III (p ≤ 0.05), ^#^Statistically significant differences between group II and group IV (p ≤ 0.05), ^&^Statistically significant differences between group I and group IV (p ≤ 0.05), ^¶^Statistically significant differences between group III and group IV (p ≤ 0.05).

### Effect of intraperitoneal administration of CYP on urinary bladder activity in normal rats

**Figure 2:**
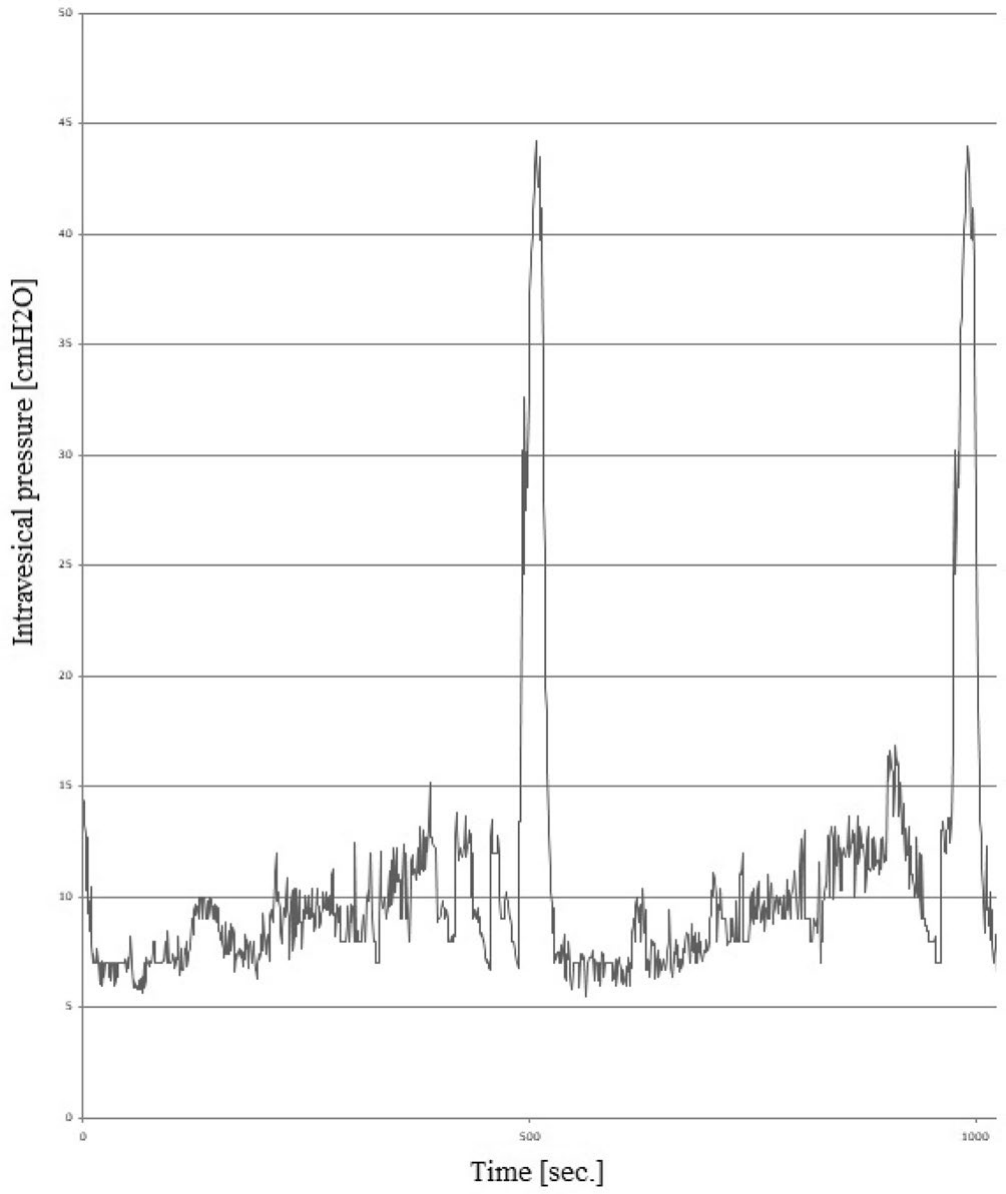
Cystometrogram in rats with cyclophosphamide (CYP)-induced detrusor overactivity. The figure shows a 1000-s interval (horizontal axis). The vertical axis is the intravesical pressure (range: 0–50 cmH_2_O).

Intraperitoneal administration of CYP induced HC leading to detrusor overactivity (DO) (n = 15) (Fig. 2). Cystometry results showed an increase of BP (approx. 131%), ANVC (approx. 248%), FNVC (approx. 2126%), AUC (approx. 248%), and DOI (approx. 1240%). Additionally, a significant decrease of TP (approx. 20%), ICI (approx. 54%), BC (approx. 29%), VV (approx. 47%), and VTNVC (approx. 50%) were recorded (Table 2). No statistical differences of MVP and PVR were observed.

**Table 2:** Cystometric parameters of experimental groups.

Cystometric parameters	Experimental groups				p
	I control (n = 15)	II CYP-induced DO (n = 15)	III PCE (n = 15)	IV CYP-induced DO + PCE (n = 15)	
BP (cmH_2_O)	3.2 ± 0.20	7.4 ± 0.50*	3.2 ± 0.19^§^	4.0 ± 0.28^#^	p < 0.0001* p < 0.0001^§^ p < 0.0001^#^
TP (cmH_2_O)	8.0 ± 0.29	6.4 ± 0.31*	7.4 ± 0.33	8.3 ± 0.32^#^	p = 0.0037* p = 0.0004^#^
MVP (cmH_2_O)	47 ± 1.6	43 ± 2.1	44 ± 1.7	40 ± 1.5	NS
ICI (sec)	1096 ± 37	503 ± 23*	1082 ± 33	956 ± 26^#,&^	p < 0.0001* p < 0.0001^#^ p = 0.0095^&^
BC (ml/cmH_2_O)	0.24 ± 0.013	0.17 ± 0.009*	0.23 ± 0.016^§^	0.21 ± 0.007^#^	p = 0.0004* p = 0.0010^§^ p = 0.0289^#^
VV (ml)	1.0 ± 0.040	0.53 ± 0.030*	0.95 ± 0.043^§^	0.93 ± 0.039^#^	p < 0.0001* p < 0.0001^§^ p < 0.0001^#^
PVR (ml)	0.052 ± 0.009	0.050 ± 0.004	0.075 ± 0.005^§^	0.076 ± 0.006^#^	p = 0.0488^§^ p = 0.0402^#^
VTNVC (%)	78 ± 2.3	39 ± 2.9*	75 ± 3.3^§^	67 ± 3.1^#,&^	p < 0.0001* p < 0.0001^§^ p < 0.0001^#^ p = 0.0344^&^
ANVC (cmH_2_O)	2.3 ± 0.078	8.0 ± 0.420*	2.4 ± 0.099^§^	3.5 ± 0.240^#,&,¶^	p < 0.0001* p < 0.0001^§^ p < 0.0001^#^ p = 0.0056^&^ p = 0.0183^¶^
FNVC (times/filling phase)	0.31 ± 0.029	6.9 ± 0.420*	0.23 ± 0.018^§^	1.7 ± 0.160^#,&,¶^	p < 0.0001* p < 0.0001^§^ p < 0.0001^#^ p = 0.0003^&^ p < 0.0001^¶^
AUC (cm H_2_O/s)	28 ± 1.50	41 ± 1.20*	24 ± 1.50^§^	24 ± 0.96^#^	p < 0.0001* p < 0.0001^§^ p < 0.0001^#^
DI/DOI (cmH_2_O/ml)	25 ± 1.8	335 ± 19*	29 ± 1.9^§^	106 ± 3.8^#,&,¶^	p < 0.0001* p < 0.0001^§^ p < 0.0001^#^ p < 0.0001^&^ p < 0.0001^¶^

*Statistically significant differences between group I and group II (p ≤ 0.05), ^§^Statistically significant differences between group II and group III (p ≤ 0.05), ^#^Statistically significant differences between group II and group IV (p ≤ 0.05), ^&^Statistically significant differences between group I and group IV (p ≤ 0.05), ^¶^Statistically significant differences between group III and group IV (p ≤ 0.05).

### Effect of oral administration of PCE on urinary bladder activity in normal rats

Cystometric parameters showed that oral administration PCE in dose of 500 mg/kg had no influence on storage and voiding phase of micturition cycles (n = 15). As compared to naïve animals we found no statistically significant changes in cystometry (Table 2).

### Effect of oral administration of PCE on urinary bladder motor activity in rats with CYP-induced HC

**Figure 3:**
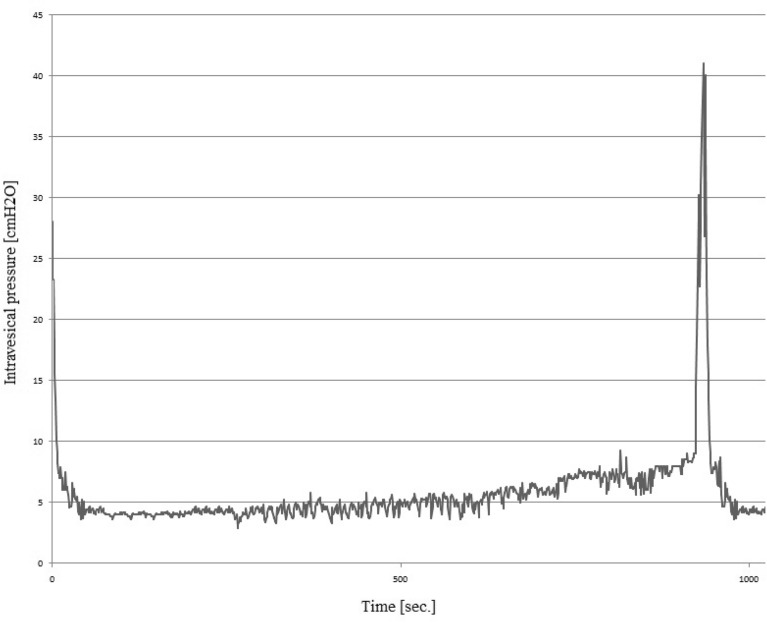
Cystometrogram in rats with cyclophosphamide (CYP)-induced detrusor overactivity after *Potentilla chinensis extracts* administration. The figure shows a 1000-s interval (horizontal axis). The vertical axis is the intravesical pressure (range: 0–45 cmH_2_O).

Our study revealed that oral intake of PCE in dose of 500 mg/kg attenuates the severity of CYP-induced HC (n = 15) (Fig. 3). The DO which is typical for HC is diminished in the response to PCE administration. In comparison with CYP-induced HC animals we reported a statistical significant decrease of BP (46%), ANVC (approx. 56%), FNVC (approx. 75%), AUC (approx. 41%), and DOI (approx. 68%). Furthermore, we observed a statistical significant increase of ICI (approx. 90%), BC (approx. 23%), VV (approx. 75%), PVR (approx. 52%), and VTNVC (approx. 72%), and TP (approx. 30%) (Table 2). No statistical differences of MVP was obtained.

### Effect of intraperitoneal administration of CYP on biochemical profile of urinary bladder urothelium and detrusor muscle

**Figure 4:**
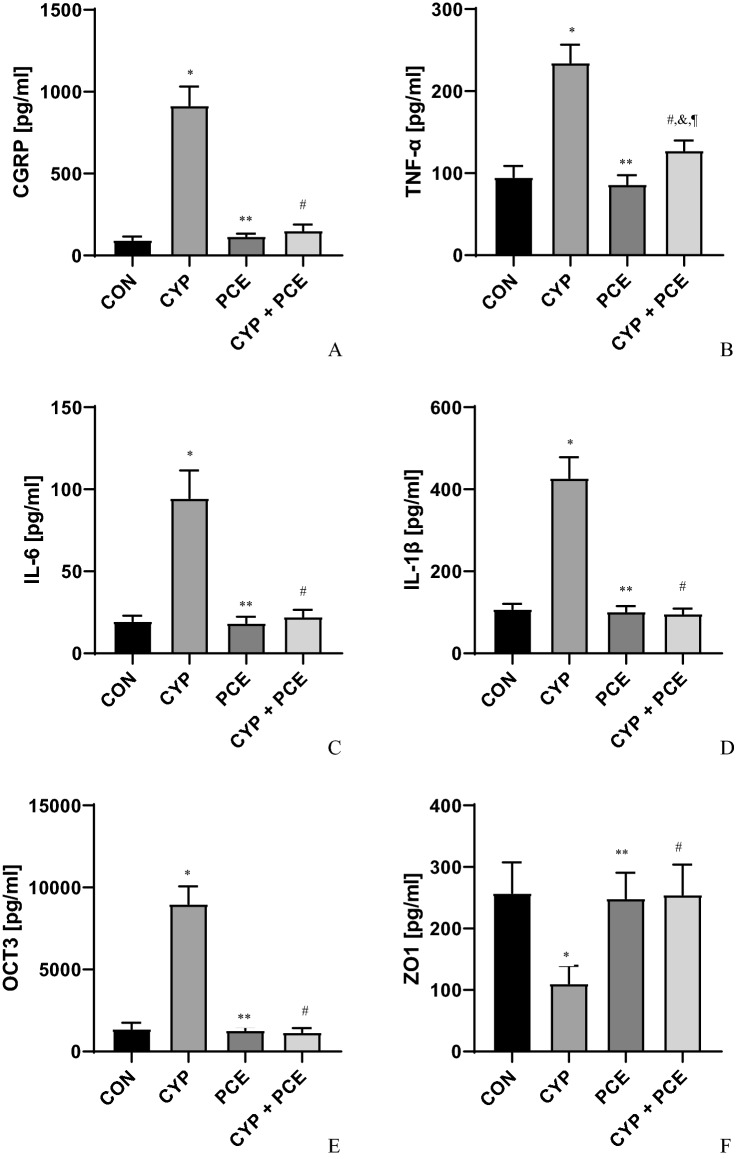
The influence of cyclophosphamide (CYP) and *Potentilla chinensis* aqueous extract (PCE) in naïve rats and PCE in rats with CYP-induced cystitis on the level of (**A**) CGRP, (**B**) TNF-α, (**C**) IL-6, (**D**) IL-1β, (**E**) OCT3, (**F**) ZO-1 in urothelium.

**Figure 5:**
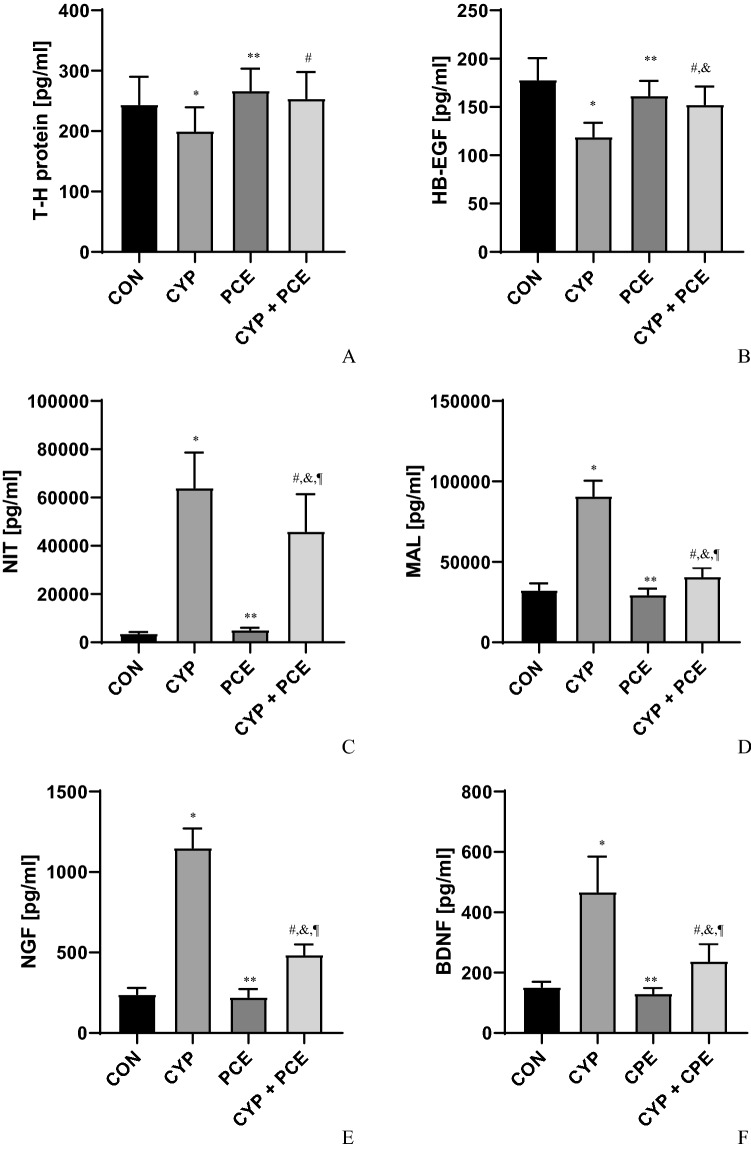
The influence of cyclophosphamide (CYP) and *Potentilla chinensis* aqueous extract (PCE) in naïve rats and PCE in rats with CYP-induced cystitis on the level of (**A**) T–H protein, (**B**) HB-EGF, (**C**) NIT, (**D**) MAL, (**E**) NGF, (**F**) BDBF in urothelium.

In urothelium, a significant increase in the concentration of CGRP, TNF-α, IL-6, IL-1β, OTC_3,_ NIT, and MAL have been observed following the intraperitoneal administration of CYP (n = 15). Additionally, the level of T-H protein, HB-EGF, and ZO1 decreased significantly within urothelium (n = 15). CYP-treated animals were characterized by increased level of ROCK1 and VAChT in detrusor muscle (n = 15) (Figs. 4, 5, Table 3).

**Table 3:** Biochemical parameters of experimental groups.

Biochemical parameters	Experimental groups				p
	I control (n = 15)	II CYP-induced DO (n = 15)	III PCE (n = 15)	IV CYP-induced DO + PCE (n = 15)	
CGRP (pg/ml)	94 ± 5.5	916 ± 30*	117 ± 4.3^§^	152 ± 9.7^#^	*p < 0.0001 ^§^p < 0.0001 ^#^p < 0.0001
TNF-α (pg/ml)	95 ± 3.6	235 ± 5.7*	86 ± 2.9^§^	128 ± 3.2^#,&,¶^	*p < 0.0001 ^§^p < 0.0001 ^#^p < 0.0001 ^&^p < 0.0001 ^¶^p < 0.0001
IL-6 (pg/ml)	20 ± 0.9	95 ± 4.3*	18 ± 1.0^§^	22 ± 1.1^#^	*p < 0.0001 ^§^p < 0.0001 ^#^p < 0.0001
IL-1β (pg/ml)	108 ± 3.4	427 ± 13*	102 ± 3.5^§^	97 ± 3.1^#^	*p < 0.0001 ^§^p < 0.0001 ^#^p < 0.0001
OCT3 (pg/ml)	1386 ± 94	8999 ± 278*	1295 ± 54^§^	1166 ± 68^#^	*p < 0.0001 ^§^p < 0.0001 ^#^p < 0.0001
T–H protein	244 ± 12	200 ± 10*	267 ± 9.4^§^	254 ± 11^#^	*p = 0.0269 ^§^p = 0.0003 ^#^p < 0.0044
HB-EGF	178 ± 5.7	119 ± 3.7*	162 ± 3.9^§^	153 ± 4.8^#,&^	*p < 0.0001 ^§^p < 0.0001 ^#^p < 0.0001 ^&^p = 0.0013
NIT	3720 ± 155	64,070 ± 3753*	5189 ± 210^§^	46,051 ± 3947^#,&,¶^	*p < 0.0001 ^§^p < 0.0001 ^#^p = 0.0001 ^&^p < 0.0001 ^¶^p < 0.0001
MAL	32,570 ± 1065	90,883 ± 2458*	29,713 ± 968^§^	40,984 ± 1391^#,&,¶^	*p < 0.0001 ^§^p < 0.0001 ^#^p < 0.0001 ^&^p = 0.0023 ^¶^p < 0.0001
ZO1 (pg/ml)	257 ± 13	111 ± 7.5*	249 ± 11^§^	253 ± 13^#^	*p < 0.0001 ^§^p < 0.0001 ^#^p < 0.0001
**Detrusor muscle**					
ROCK1 (pg/ml)	1648 ± 66	4052 ± 153*	1913 ± 78^§^	2197 ± 72^#,&^	*p < 0.0001 ^§^p < 0.0001 ^#^p < 0.0001 ^&^p = 0.0013
VAChT (pg/ml)	5194 ± 277	45,371 ± 1955*	6103 ± 337^§^	9043 ± 358^#,&^	*p < 0.0001 ^§^p < 0.0001 ^#^p < 0.0001 ^&^p = 0.0469
**Urine**					
BDNF (pg/ml)	152 ± 4.5	468 ± 30*	131 ± 4.7^§^	238 ± 15^#,&,¶^	*p < 0.0001 ^§^p < 0.0001 ^#^p < 0.0001 ^&^p = 0.0040 ^¶^p < 0.0001
NGF (pg/ml)	241 ± 10	1151 ± 31*	223 ± 13^§^	487 ± 16^#,&,¶^	*p < 0.0001 ^§^p < 0.0001 ^#^p = 0.0001 ^&^p < 0.0001 ^¶^p < 0.0001

*Statistically significant differences between group I and group II (p ≤ 0.05), ^§^Statistically significant differences between group II and group III (p ≤ 0.05), ^#^Statistically significant differences between group II and group IV (p ≤ 0.05), ^&^Statistically significant differences between group I and group IV (p ≤ 0.05), ^¶^Statistically significant differences between group III and group IV (p ≤ 0.05).

### Effect of oral administration of PCE on biochemical profile of urinary bladder in rats with CYP-induced HC

**Figure 6:**
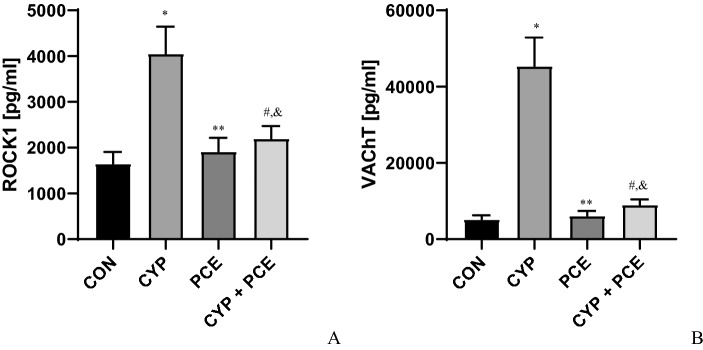
The influence of cyclophosphamide (CYP) and *Potentilla chinensis* aqueous extract (PCE) in naïve rats and PCE in rats with CYP-induced cystitis on the level of (**A**) ROCK1, and (**B**) VAChT in detrusor muscle.

In comparison with CYP-treated animals, the PCE administration in dose of 500 mg/kg prevented the occurrence of an increased change in the biochemical profile in response to CYP (n = 15). In comparison with CYP-induced HC, PCE diminished the urothelial concentration of CGRP, TNF-α, IL-6, IL-1β, OTC_3,_ NIT, and MAL (n = 15). In contrast the urothelial level of T-H protein, HB-EGF, and ZO1 increased significantly (n = 15). In CYP-induced HC animals, the oral supply of PCE reduced the ROCK1 and VAChT concentration in detrusor muscle (n = 15) (Figs. 4, 5, 6, Table 3).

### Effect of intraperitoneal administration of CYP and oral administration of PCE on biochemical profile of urine samples

Intraperitoneal administration of CYP caused an increased concentration of BDNF and NGF in the urine samples (n = 15). Furthermore, a reduction of urine BDNF and NGF concentration was observed in response to the PCE (dose: 500 mg/kg) used (n = 15) (Fig. 5, Table 3).

## Discussion

The observations so far have shown that CYP has an effect on the lower urinary tract, and in particular on the urinary bladder. Well, chemotherapy based on CYP usually lead to structural and functional changes in the urinary bladder due to hemorrhagic cystitis (HC), affecting the quality of life of patients during the course of chemotherapy. Thus, the search for methods to reduce the “toxic” effect of CYP on the urinary bladder is extremely important in everyday clinical practice. Previous studies revealed that cumulative dose of CYP is one of the most important predictor for hemorrhagic cystitis development^23^. Mesna is commonly used in the prevention of HC^24^. Potentilla plants reduce a pro-oxidative activity with concomitant intensify an anti-oxidative activity^25^. Thus, the purpose of our study was to investigate the attenuation effect of *Potentilla chinensis* aqueous extract on urinary bladder function in CYP-induced hemorrhagic cystitis in rat model. Moreover, the biochemical profile changes within urothelium, detrusor muscle, and in the urine were determined.

Different *Potentilla* species have an anti-cancer effect, which has been confirmed by scientific research. Research to date has shown that *Potentilla chinensis* aqueous extract has a cytotoxic effect on cancer cells in in vitro studies. Liu et al.^12^ revealed that *Potentilla chinensis* aqueous extract due to triterpenes constituents has the cytotoxic activity. *Potentilla* reptans exhibited also high anti-proliferative activity against MCF-7 cells whilst *Potentilla* speciosa had weak to moderate activity against both of A549 and MCF-7 cell lines^26^. The above facts allow us to conclude that *Potentilla chinensis* aqueous extract does not have the opposite effect to the cyclophosphamide. So far, no data are available describing the occurrence of an interaction between *Potentilla chinensis* aqueous extract and CYP.

CYP treatment is associated with lower urinary tract dysfunction including reduction of micturition voiding pressure, intercontraction intervals and functional urinary bladder capacity, and also an increase of non-voiding contraction frequency^27^. These dysfunctions are related to HC due to CYP administration. Previous studied on animal models revealed that CYP administration (in single or repeated doses) caused inflammatory response leading to macroscopic and microscopic changes within urinary bladder wall as follow: (1) mucosal abrasion, (2) oedema, (3) haemorrhages, and (4) increased infiltration of inflammatory cells (neutrophils, mononuclear cells)^28^. Thus an important observation during our research was that PCE diminish the severity of urinary bladder wall oedema due to CYP administration.

Our results show that CYP generates detrusor overactivity (DO) as reflected in the cystometry. CYP caused an increase of BP, ANVC, FNVC, AUC, and DOI. Additionally a decrease of TP, ICI, BC, VV, and VTNVC was observed. The valuable insights of the study is that PCE attenuates the severity of DO induced by intraperitoneal administration of CYP. In turn, PCE has no influence on urinary bladder function in healthy conditions. In CYP-induced DO, PCE administration caused an increase in TP, ICI, BC, VV, VTNVC, and PVR. Moreover a decrease of BP, ANVC, FNVC, AUC, and DOI were recorded. Cystometric evaluation shows that PCE suppress the excitability, and basal tone of detrusor during storage. Also, the PCE do not affect the detrusor contraction amplitude initiating voiding (no significant changes of MVP). Valuable insight is related with a decrease in TP, and VTNVC on the PCE administration due to suppressive effect of PCE on afferent nerves. The modulating effect of PCE on afferent innervation increase the BC and VV providing more effective urine storage.

The role of several cytokines in pathogenesis of CYP-induced HC were described^29^. IL-1β is a pivotal cytokine that triggers the immune response and stimulates the pro-inflammatory response (via TNF α and IL-6)^30^. Previous data clearly indicate that IL-1β, IL-6, TNF-α, NIT, and MAL are indicators and mediators of inflammation^31^. In CYP-induced HC an increased level of IL-1β, and IL-6 was observed^32^. Our experiment showed that CYP increases the concentration of CGRP, TNF-α, IL-6, IL-1β, OTC_3,_ NIT, and MAL (in urothelium). However, the administration of PCE diminished the severity of CYP-induced HC through inhibition of CGRP, TNF-α, IL-6, IL-1β, OTC3, NIT, and MAL. Similarly, Gomes et al.^33^ study revealed that anti IL-1 β or anti-TNF-α serum in CYP-induced HC attenuates the macroscopic changes within urinary bladder wall (e.g. mucosal erosion, hemorrhage, oedema, and ulcerations). Moreto et al.^34^ pointed that oxidative stress and chronic low-grade inflammation may promote a formation of byproducts such as MAL during the process of lipid peroxidation. Tamm–Horsfall proteins are thought to be a crucial element of the lower urinary tracts’ defense, which is active in all inflammatory conditions (e.g. IC/PBS)^35^. In our study PCE proved to be an anti-inflammatory agent by the elevation of T–H protein levels that were lowered in the animals with CYP-induced HC.

It is worth noting that CYP leads to oxidative stress mainly through inhibition of antioxidant enzymes^36^. Calcitonin-gene related peptide (CGRP) and substance P (SP) play a distinct role in the urinary bladder dysfunction after CYP administration due to neurogenic inflammation development^37^. In our study we observed a significant decrease in CGRP in PCE-treated rats animals (916 ± 30 pg/ml in the CYP group and 152 ± 9.7 in the CYP plus PCE group).

CYP increases the permeability of urothelium due to lowered concentration of ZO1 and occludin^38^. ZO1 is a link between tight junction proteins and cytoskeleton, playing an important role in the paracellular barrier^39^. In our CYP-treated animals we observed a decreased level of ZO1 within urothelium. Contrary, in animals with CYP-induced hemorrhagic cystitis, a restoration of ZO1 was observed after PCE administration.

Likewise, we observed PCE diminished the severity of DO in animals with CYP-induced HC through inhibition of OTC3, ROCK1, and VAChT. Hanna-Mitchell et al.^40^ described that urotelium expresses organic cation transporter OTC3 which participates in acetylocholine (Ach) release. In our study we revealed that PCE diminished the level of OTC3 within urothelium in PCE-treated animals with CYP-induced HC. The blockade of ROCK-dependent pathway suppress the inflammatory response. ROCK inhibitors suppress the inflammatory response^16^. Also ROCK is linked to acetylocholine receptor—dependent transduction pathway^41^. In our experiment ROCK-pathway inhibition by PCE ameliorates DO in rats with CYP-induced HC.

CYP administration stimulates (via vanilloid receptors activation) the release of SP, CGRP, and VAChT. Ach transporter (VAChT) attends in the Ach release processes in non-neuronal cells. Urinary bladder is innervated by VAChT-, and CGRP-positive afferent nerves^42^. In CYP-induced HC we observed increased level of VAChT. In turn, the PCE decreased the level of VAChT within detrusor muscle suppressing the detrusor contractility activity (reduction of ANVC, FNVC, AUC, and DOI). Additionally, VAChT-dependent pathway modulation by PCE seems to promote relaxation of the detrusor muscle (reduction of BP, and also increment of TP, BC and ICI). Based on the above mentioned results it can be stated that PCE affect the urinary bladder activity in two ways, such as (1) impact on muscle cells activity (direct action), and (2) anti-inflammatory action through the inhibition of neurogenic inflammation (subsequent action). Therefore PCE may become an interesting alternative to antimuscarinics in DO therapy, as well as in supportive treatment of urinary bladder dysfunction in patients who undergo CYP-based chemotherapy.

Patients with interstitial cystitis present a decreased concentration of HB-EGF in urine^43^. HB-EGF play role in proper wound healing (e.g. epithelialization and wound contraction)^44^. Similarly, we was reported the same changes in CYP-treated animals, while a reverse change was found after PCE intake.

Some neurotransmitters (e.g. NGF and BDNF) are released from urotelium and smooth muscle cells. NGF and BDNF modulate the activity of afferent nerves in urinary bladder^45^. We observed that CYP administration increase the concentration of NGF and BDNF in the urine samples. Contrary, PCE reduce the concentration of these biomarkers. Our results are consistent with the results of clinical studies. Antimuscarinics and onabotulinum toxin A decrease the level of NGF and BDNF in urine samples of patients with overactive bladder^46,47^.

It should be noted that bioactive compounds isolated from *Potentilla chinensis* extract so far (e.g. trans-tiliroside, polysaccharides, etc.), which may be responsible for the antioxidant and anti-inflammatory effects of *Potentilla chinensis* extract. Therefore, it is reasonable to conduct further research aimed at defining the compounds listed which present the strongest antioxidant and anti-inflammatory effects.

## Conclusions

In conclusion, our results revealed that PCE in dose of 500 mg/kg attenuates DO in case of CYP-induced HC. The potential mechanisms of the action of PCE in the urinary bladder seems to be multifactorial and complex. PCE may inhibit the release of transmitters from afferent and efferent fibres innervating urinary bladder. The potential properties of PCE appear to be important in terms of preventing of oxidative stress-dependent dysfunction of urinary bladder. PCE may become a potential supportive treatment in addition to typical pharmacotherapy with proven effect in daily clinical practice, especially in patient to whom CYP-based chemotherapy is used.
